# When Recurring Infections Mask an Atypical Presentation of Inflammatory Bowel Disease (IBD): A Re-Visitation and Literature Review

**DOI:** 10.7759/cureus.20215

**Published:** 2021-12-06

**Authors:** Daniel T Gildea, William Davis, Natalie Dapas, Ahmad Al Nakshabandi, Lakshmi Krishnan

**Affiliations:** 1 Internal Medicine, MedStar Georgetown University Hospital, Washington, DC, USA; 2 Gastroenterology, MedStar Georgetown University Hospital, Washington, DC, USA

**Keywords:** ulcerative colitis, crohn's disease, inflammatory bowel disease, pg, extra-intestinal manifestations, pyoderma gangrenosum, ibd

## Abstract

Pyoderma gangrenosum (PG) is an uncommon and severe extra-intestinal manifestation (EIM) of inflammatory bowel disease (IBD). Head or scalp involvement in this condition is exceedingly rare. Approximately one-third of presentations can be precipitated by skin trauma or infection, a phenomenon known as pathergy. These ulcers develop acutely, do not necessarily correlate with IBD activity, and can precede IBD diagnosis. Here, we present an atypical presentation of PG that became a cornerstone finding in the subsequent diagnosis of IBD.

## Introduction

This case report discusses a series of atypical soft-tissue infections which ultimately led to a diagnosis of pyoderma gangrenosum (PG) in the setting of inflammatory bowel disease (IBD), drawing attention to a lesser-known and more unexpected presentation of IBD. This article aims to additionally delve deeper into PG, both in terms of diagnosis and treatment as well as its relationship with IBD. Here, we analyze the foundation of literature upon which our current understanding of PG is built [1-4], diagnostic indicators and its association with IBD [1,5-6], and emerging treatments [7,8]. Through this discussion, a holistic picture of PG, its manifestations, its association with IBD, and potential treatment options, is formed. While there is much more to understand, our report aims to shed some more light on PG and draw together recent advances into a succinct, up-to-date description. This report represents an update to a previously published case by Davis, William D., et al entitled "S2445 When Recurring Infections Mask an Atypical Presentation of Inflammatory Bowel Disease."

## Case presentation

**Figure 1:**
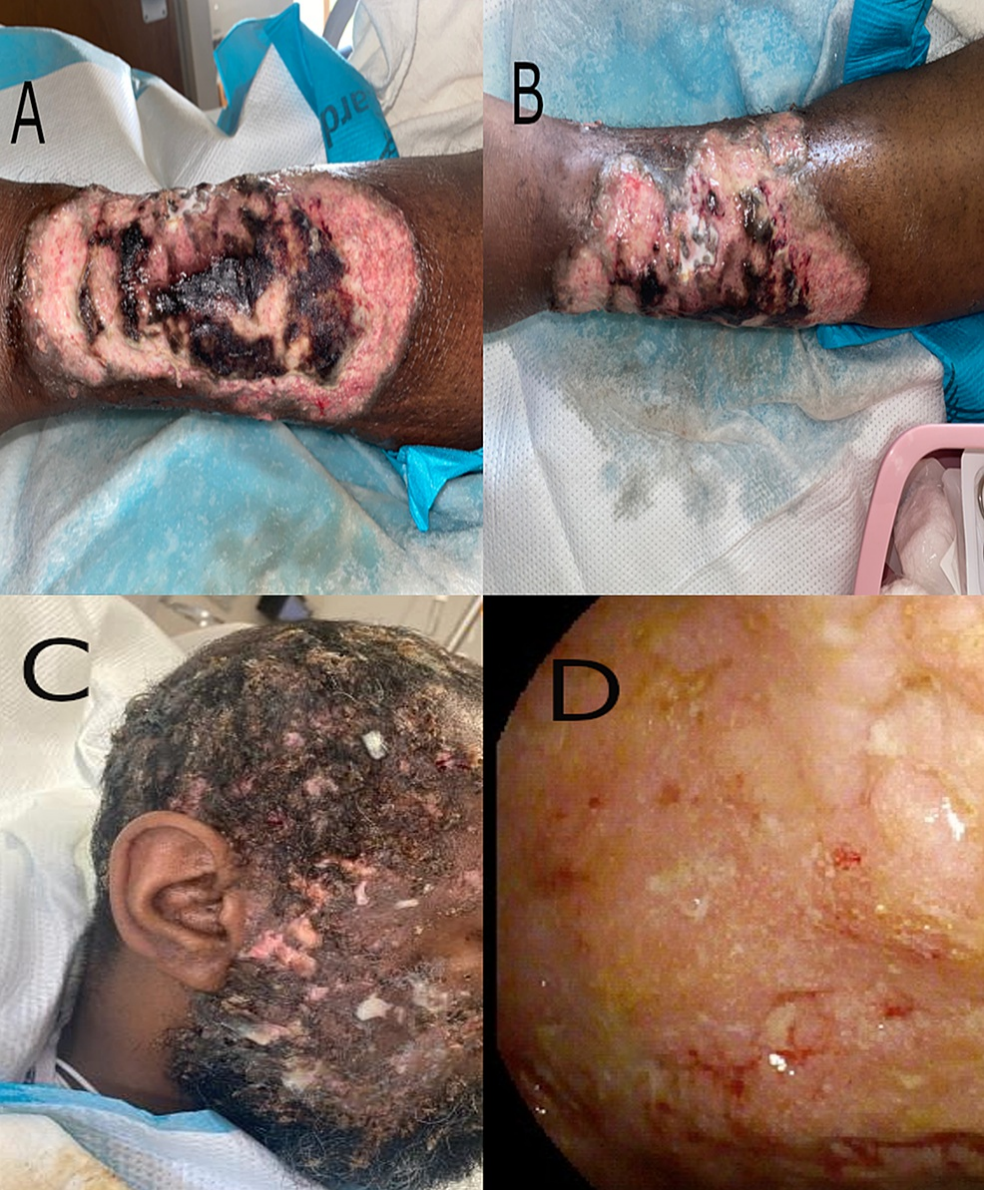
Pyoderma gangrenosum (PG) lesions and endoscopy findings. A, B: Lesions identified on patient's left lower extremity. C: Facial and scalp lesions identified on the patient. D: Sigmoidoscopy findings showing rectosigmoid mucosa with ulcerations, congestion, erythema, granularity, and loss of vascularity.

A 35-year-old male with prior diagnosis of recurrent, diffuse soft tissue infections presented for management of presumed sepsis due to soft tissue infection. Two weeks prior to presentation, the patient developed a painful and purulent left lower extremity ulcer (Figure 1A, B) followed by worsening of facial and scalp ulcerations (Figure 1C). 

Of note, the patient stated that his lesions started on his face in January 2020; he went four months without evaluation due to lack of insurance until they spontaneously regressed. He remained without further recurrence until March 2021 (about three months prior to the present admission), when they recurred, ultimately leading to hospitalization at that time where he improved with IV antibiotics. During that admission, he experienced some abdominal pain, leading to a CT abdomen and pelvis which showed circumferential sigmoid and rectal wall thickening concerning for colitis. After that discharge, again three months before the present admission, the patient was lost to follow-up and did not undergo endoscopy despite the recommendation to do so. 

On present admission, the patient was febrile to 39.4˚C, WBC count of 33k/ul and hemoglobin of 6.1 g/dl. Because of this low hemoglobin, and because he had not undergone the previously recommended outpatient endoscopy for the CT findings, sigmoidoscopy was performed showing diffuse erosive gastritis. Wound cultures of his left lower extremity (Figure 1A, B) grew staphylococcus aureus, and he was started on IV antibiotics. Initial concerns for IBD were based on the prior CT imaging, the ulcers on his leg and scalp which were grossly concerning for PG, and a three-year history of "off-and-on" watery diarrhea with occasional dark brown to black stools elicited after admission. Laboratory work-up is summarized in Table 1. Sigmoidoscopy showed cobblestoning with non-bleeding mucosal ulcerations and sigmoid colon biopsies demonstrated severe active colitis (Figure 1D). Skin biopsy of his left cheek demonstrated disrupted follicular infundibula with adjacent neutrophil-rich mixed infiltrate and adjacent cicatrix on a fibrotic background dermis, non-specific findings which could represent early pyoderma gangrenosum, infection, hidradenitis, acne conglobata, or other follicular disorders. Patient was started on steroid therapy with the improvement of both diarrhea and inflammatory markers. The patient was discharged on oral steroids and planned to follow up for initiation of biological therapy; he is now managed outpatient on ustekinumab.

**Table 1:** Relevant labs. Workup elucidated significant bowel inflammation (elevated ESR, CRP, fecal calprotectin, elevated WBC), with no infectious or clear autoimmune etiology identified (negatives). WBC: white blood cells; HIV: human immunodeficiency virus; HCV: hepatitis C virus; HBV: hepatitis B virus; ANA: Antinuclear antibodies; dsDNA: double-stranded DNA; ANCA: antineutrophil cytoplasmic antibodies; MPO: myeloperoxidase; PR3: proteinase 3.

	5/25/21	5/31/31	6/7/21	6/10/21
C-reactive protein (nl 0-10mg/L)	270	36	28	
Sedimentary rate (nl 0-15mm/hr)	127			
Fecal calprotectin (nl 10-50mcg/gm)	>3000			
WBC (nl 4-10.8k/ul)	33	12.2	14.5	10.5
HIV	Negative			
HCV	Negative			
HBV Core/sAg	Negative			
ANA	Negative			
dsDNA	Negative			
ANCA	Negative			
MPO	Negative			
PR3	Negative			
Clostridium difficile	Negative			
Ova and parasite	Negative			
Stool culture	Negative			

## Discussion

Discussion and literature review

IBD is associated with a broad array of extra-intestinal manifestations (EIM). Some of these findings are likely associated with the inflammatory state of the disease itself, while other findings may be related to the autoimmune susceptibility of the patients themselves, or the treatment regimens used to treat these disorders [1]. These extra-intestinal manifestations include arthritis, erythema nodosum, aphthous stomatitis, uveitis, ankylosing spondylitis, Henoch-Schoenlein purpura, primary sclerosing cholangitis, primary biliary cholangitis, arteritis, polymyositis, and many more, including pyoderma gangrenosum [1]. These EIM can be essential in supporting the diagnosis of IBD, as demonstrated in the preceding case report.

PG is an ulcerative dermatosis with an incidence of about 6 per 100,000, although given difficulties in diagnosis the true incidence is yet unknown [2]. The pathophysiology is also not fully elucidated, though there appears to be a multifactorial process at play. Histopathogically, PG lesion biopsies have been shown to contain neutrophilic abscesses, suppurative inflammation with dermal edema, and elevated levels of several pro-inflammatory cells and cytokines including IL-6, as well as a reduced ratio between T regulatory cells (which help prevent inflammation) and Th-17 cells. These biopsy findings implicate neutrophil dysfunction along with dysfunction in both the innate and adaptive immune system processes in the production of PG lesions [2]. Our biopsy report did not comment on cytokines, and otherwise differs from the above findings by its absence of neutrophilic abscesses and dermal edema, although a neutrophil-rich infiltrate was noted. This underscores the importance of clinical correlation with such non-specific findings.

About 50% of PG cases are associated with an underlying disorder, or, in some cases, pharmacological therapies [3]. Inflammatory bowel disorders - both ulcerative colitis and Crohn’s disease, account for 20-30% of these cases; however, only ~3% of patients with IBD will actually develop PG [3]. Other associated disease states include hepatitis C, seronegative rheumatoid arthritis, spondylitis, and various lymphoproliferative disorders including leukemia and lymphoma [3]. Implicated drugs include propylthiouracil, pegfilgastrim, and gefinib [3]. The role of genetics has been increasingly investigated, with multiple PG-associated genetic syndromes (PAPA, PASH, etc) found to be associated with pro-inflammatory mutations in a subset of genes [2]. Some reports in the literature also describe familial PG in the setting of disease, surgery, and unprovoked findings [2].

While it most often occurs after initial symptoms present, PG can occur at any stage of the IBD disease process, ranging from before the presentation of colitis to even after colectomy [4]. It seems to have a closer association with ulcerative colitis (UC) than with Crohn’s, and while disease exacerbations can correlate with worsening PG lesions, there does not seem to be a strong connection per se with colitis activity. For example, even when colitis symptoms become quiescent, PG can continue to persist [4].

Clinically, PG can present with a variety of findings. The most common presentation is classical PG, a painful, rapidly progressing ulcer with a violaceous, undermined edge; this accounts for about 85% of cases [5]. Other findings are less common and include bullous PG - associated with hematological malignancies - which presents (often on the arms) with waves of painful, coalescing vesicles and bullae. Pustular PG - associated with IBD flares - presents in extensor regions with painful pustules overlying an erythematous base. Granulomatous superficial PG, often found without underlying illness, is slowly progressing, with verrucous and ulcerating lesions. Peristomal PG, associated with the well-known pathergy response of the PG process, is often found in IBD patients in irritated areas, particularly stomas. Finally, the rare malignant pyoderma - a process not associated with systemic disease - presents with destructive ulceration of the upper body, including the head and neck area [5]. The most common ulcer finding is on the lower legs, though ulcers can be found anywhere on the body; pathergy, the tendency for PG ulcers to form in areas of even minor trauma, can play a role in this and may often lead to misdiagnosis. One recent systematic case review identified 60 reports of PG patients. Of these, 29 (48.4%) had findings on the lower limbs and 19 (31.7%) had findings on the upper limbs and torso. In comparison, only 3 (5.0%) had facial lesions [6], underscoring its rarity in presentation and its potential for a difficult diagnosis, as in this manuscript’s reported case.

Definitive diagnostic criteria of PG remain elusive. As PG is often considered a diagnosis of exclusion, clinical judgment and a thorough review of patient symptoms is of critical importance. Given the variety of potential presentations, as well as the varied (or absent) associated disease states, establishing the diagnosis can be difficult. In light of this, attempts have been made to define specific diagnostic criteria for PG, most recently in 2018. This Delphi Consensus of international experts, after deliberation, produced a validated list of eight minor and one major criteria. The eight minor criteria include exclusion of infection, pathergy, known IBD or inflammatory arthritis, history of papule, pustule, or vesicle ulcerating within four days of first appearance, peripheral edema, undermining border, and tenderness of ulceration, multiple ulcers including 1 on the anterior lower leg, cribriform (“wrinkled paper”) scars at healed ulcer sites, and a decrease in ulcer size within one month of starting immunosuppressive medications [7]. The one major criteria was ulcer edge biopsy demonstrating a neutrophilic infiltrate. This 2018 paper’s ROC analysis determined that the presence of four out of the eight minor criteria, in addition to the one major criterion, maximized discrimination [7]. Our patient did meet the major criterion, while additionally meeting at least the minor criteria of tender ulcerations and multiple ulcers. Not all criteria could be evaluated at that time, such as a decrease in ulcer size with immunosuppressive medication or a known IBD diagnosis. Unfortunately, the association of PG with pathergy - which is in fact one of the minor criteria - can lead to hesitance to biopsy due to concern of worsening the ulcer through biopsy-associated trauma.

Like diagnosis, management of PG can be challenging. Intensive wound care in combination with supportive care is an important first-line treatment. This can be used in combination with topical steroids and tacrolimus. In severe cases, systemic corticosteroids, sometimes in combination with cyclosporine or other immunosuppressive drugs such as methotrexate, mycophenolate mofetil, and azathioprine, are used [5]. Biologic therapies have been increasingly investigated for PG treatment. A recent semi-systematic review of TNF-alpha inhibitors in a set of 356 patients with PG noted an 87% response rate to TNF-a inhibitors, 67% of which was a complete response. There was no significant difference between the usage of infliximab, etanercept, or adalimumab found [8]. A similar study on 81 PG patients treated with interleukin inhibitors demonstrated a 79% response to ustekinumab, 64% for canakinumab, and 59% for anakinra, with 71%, 55%, and 38% complete response rates, respectively. This led to a 70% response rate and 57% complete response rate for interleukin inhibitors as a class; the study was not able to differentiate significantly among treatment types due to data heterogeneity [9].

## Conclusions

This case emphasizes the importance of the history and physical exam in the evaluation of a patient. This individual initially presented with recurrent spontaneous skin ulcers and infections; however, the history of recurrent diarrhea in addition to the CT imaging demonstrating findings consistent with colitis moved the workup beyond infectious etiologies to the ultimate diagnosis and treatment of PG and IBD. This recognition led to the prompt, appropriate management of both the skin lesions and the underlying inflammatory bowel disease.
